# The Effects of Mild Disturbances on Sleep Behaviour in Laying Hens

**DOI:** 10.3390/ani13071251

**Published:** 2023-04-04

**Authors:** Endre Putyora, Sarah Brocklehurst, Frank Tuyttens, Victoria Sandilands

**Affiliations:** 1 Department of Veterinary and Biosciences, Faculty of Veterinary Medicine, Ghent University, 9820 Merelbeke, Belgium; 2 Department of Agriculture, Horticulture and Engineering Sciences, Scotland’s Rural College (SRUC), Edinburgh EH25 9RG, UK; 3 Biomathematics & Statistics Scotland (BioSS), Edinburgh EH9 3FD, UK; 4 Animal Sciences Unit, Flanders Research Institute for Agriculture, Fisheries and Food (ILVO), 9090 Melle, Belgium

**Keywords:** welfare, recovery sleep, EEG, SWS, REM sleep, wakefulness

## Abstract

**Simple Summary:**

Farm animal welfare is important to both farmers and consumers. Sleep behaviour may offer an additional means of assessing welfare at night, where our understanding is comparatively poor. However, our present understanding of sleep in poultry is lacking. The objectives of this study were to establish a baseline for undisturbed sleep behaviour in laying hens and to then apply brief disturbances (wind, noise and light for 5 min every 30 min) and observe the subsequent effects. Sleep during lights off was comprised of two states: slow-wave sleep (SWS) and rapid eye movement (REM) sleep. Over all the types of nights, SWS constituted 58% of nighttime behaviour, and REM sleep constituted 18%, with the remaining 24% attributed to being awake. The disturbances were effective at waking laying hens when applied but had no significant carry over effects on the distributions of sleep and wakefulness behaviours into the day or next night. The patterning of sleep behaviour in laying hens is similar to other avian species, including the presence of some degree of resilience to sleep disturbances. These results further improve our understanding of this behaviour in laying hens and suggest that these short-term disturbances can be adequately compensated for within the same period shortly following disturbance.

**Abstract:**

The positive welfare of commercial animals presents many benefits, making the accurate assessment of welfare important. Assessments frequently use behaviour to determine welfare state; however, nighttime behaviours are often ignored. Sleep behaviour may offer new insights into welfare assessments. This study aimed to establish a baseline for sleep behaviour in laying hens and to then apply mild short-term disturbances and observe the subsequent effects. Twelve laying hens were divided into four batches and were surgically implanted with electroencephalogram (EEG) devices to record their brain activity. The batches were subjected to undisturbed, disturbed and recovery types of nights. Disturbed nights consisted of systematic sequences of disturbance application (wind, 90 dB noise or 20 lux light) applied one at a time for 5 min every 30 min from 21:00 to 03:00 (lights off period: 19:00–05:00). Sleep state was scored using EEG data and behaviour data from infrared cameras. Over all the types of night hens engaged in both SWS (58%) and REM sleep (18%) during lights off. When applied, the disturbances were effective at altering the amounts of wakefulness and SWS (Time × Type of Night, *p* < 0.001, *p* = 0.017, respectively), whereas REM sleep was unaltered (*p* = 0.540). There was no evidence of carry-over effects over the following day or night. Laying hens may be resilient to short-term sleep disruption by compensating for this in the same night, suggesting that these disturbances do not impact their long-term welfare (i.e., over days). Sleep behaviour potentially offers a unique means of assessing an aspect of animal welfare that, to date, has been poorly studied.

## 1. Introduction

Welfare monitoring constitutes an indispensable part of farm animal management. Good welfare provides many benefits, including improved performance (e.g., [1,2,3]) and survivability [4] as well as fulfilling both ethical and legal obligations [5]. The assessment of farm animal welfare has often been based on a combination of behavioural and physiological data. Behavioural parameters offer an animal-focused assessment of welfare which is particularly in keeping with one of the Five Freedoms, namely, the freedom to express normal behaviours [6]. The behavioural measures of welfare in a commercial context are varied. For example, studies looking at the provision of alternative diets in broiler breeders used feeding motivation as a prevalent behavioural indicator of positive welfare and a healthy level of satiety [7,8,9]. Other research has looked at the time budgets of behaviour in farm animals in relation to their wild-living relatives, such as in pigs or in domestic fowl [10,11]. Other uses of behaviour as a means to assess welfare in poultry include studies on feather pecking (e.g., [12,13,14]), perching behaviour (e.g., [15,16,17]) and preening (e.g., [18,19]).

Additionally, resting behaviour has also been used as a measure of welfare, though, often, this is as part of a broader measure of activity budgets wherein it is considered among the complete range and frequency of other common behaviours [20,21]. While such measures of the activity budget (and, by proxy, resting) can be useful indicators in the assessment of welfare in livestock or zoo animals, such as in the identification of stereotypic behaviour (see [22]), there tends to be a greater focus on observable daytime activity, with little or no consideration for nighttime sleeping behaviour. While this might be due to limitations in observing animals at night, there, nevertheless, remains a considerable knowledge gap not only in our understanding of sleep behaviour as a whole but also in its greater applications in the behavioural assessment of animal welfare.

Generally, sleep can be characterised either behaviourally or electrophysiologically (by brain activity). The broader behavioural definition of sleep is outlined by five key criteria: (1) a stereotypic or species–specific sleep posture, (2) behavioural quiescence, (3) an elevated arousal threshold, (4) rapid reversibility from this state with sufficient stimulus and (5) the presence of rebound sleep following a period of sufficient deprivation [23,24]. When looking at sleep from an electrophysiological perspective, sleep (in mammals) is divided into two main phases: rapid eye movement (REM) sleep, also referred to as paradoxical sleep, and slow-wave sleep (SWS), also referred to as non-rapid eye movement (NREM) sleep. REM sleep is characterized by low-amplitude high-frequency brain waves, while SWS is characterized by high-amplitude low-frequency waves [25,26,27]. In addition to the electrophysiological characteristics, SWS is further identified by behavioural quiescence and a reduction in muscle tone [28]. Likewise, REM sleep is further classified by rapid eye movements, complete muscle atonia, twitching of the facial muscles and extremities and irregular heart and breathing rates [23,29].

Unfortunately, despite chickens being used in one of the first electroencephalogram (EEG) sleep studies [30], the vast majority of work on sleep behaviour has been done on humans and rats (e.g., [31,32,33,34,35,36]), resulting in a comparatively poorer understanding of sleep in other groups of animals, including birds [30]. Despite this, sleep in birds is surprisingly similar to that in mammals. Birds also engage in both SWS and REM sleep, although they have significantly less REM sleep compared to mammals [37,38]. Furthermore, the muscle atonia seen in mammals is largely restricted in birds to the neck and, occasionally, the wings [39,40,41]. Birds are further unique (alongside cetaceans) in their ability to sleep uni-hemispherically, e.g., keeping one hemisphere active and the contralateral eye open at a time while the other hemisphere and eye are sleeping and closed, respectively [29,39,42,43,44,45,46,47,48]. They can also resist sleep pressure/deprivation and forego the effects of extreme deprivation entirely, if only under specific circumstances. However, under normal circumstances, birds succumb and respond to sleep deprivation in much the same way as mammals [49]. Specifically, wild migratory birds, such as sparrows (*Zonotrichia leucophrys gambelii*) and warblers (*Sylvia Borin*), have been observed to fly great distances during the night and to spend little time engaging in brief bouts of napping during the day, with seemingly no detrimental effects as a result of this deprivation. However, this resilience is only present during the migratory season [50,51,52,53]. Studies on both wild male pectoral sandpipers (*Calidris melanotos*) and great frigate birds (*Fregata minor*) have found that the need for sleep can be highly plastic at least for brief periods of time, such as during mating or migration [54,55,56].

However, our understanding of avian sleep and the exact degree of this plasticity is unknown in domestic chickens (*Gallus gallus domesticus)* and, in particular, commercially kept chickens. This is partly due to greater focus being placed on production metrics and improving performance in commercial animals rather than purely behavioural studies, such as studies looking at the behavioural time budgets of broilers in varying light conditions (e.g., [57]), studies looking at improving reproductive success (e.g., [58]) and/or studies looking to improve feed conversion efficiency (e.g., [59]). The studies that have looked at sleep in chickens have either been limited to purely behavioural observations, which—while useful—are limited in describing the full breadth of sleep, or are, in the face of ever-advancing technology, out of date [30,60,61,62]. The only studies employing EEGs in order to specifically observe the sleep behaviour of poultry were those by Ookawa and Gotoh [30], Ookawa [60] and van Luijtelaar et al. [63]. These seminal studies were fundamental in establishing the use of EEGs to study sleep. Their overall findings showed that chickens engage mostly in SWS, which is often interrupted by REM sleep [30]. REM sleep is described as only occurring at night and lasting between 5 and 30 sec per bout [30]. The few remaining papers used behavioural observations to describe the timing and duration of resting and sleeping behaviours (purely based on posture) [64] or to describe perching behaviour and perching motivation [65,66]. Moreover, work by Boerema et al. [67], using behavioural observations, found that laying hens deprived of sleep exhibited a decrease in monocular (i.e., uni-hemispheric) sleep in favour of binocular sleep. While a fair number of studies have looked at sleep behaviour in juvenile birds (e.g., [42,68,69,70]), the great discrepancy in sleep requirements and behaviour between juvenile and adult birds renders comparisons inappropriate.

It is, therefore, evident that there remains a gap when it comes to our understanding of sleep in poultry. Consequently, the aim of this study was to combine both behavioural and physiological (via EEG) observations to first establish a baseline for sleep in laying hens and to observe the short-term effects of disturbances on laying hen sleep behaviour, with the potential of using sleep as a welfare indicator in the future.

## 2. Materials and Methods

### 2.1. Animals

This study was approved by SRUC’s Animal Experiments Committee prior to beginning. A group of 19 H&N Brown Nick (brown-feathered) laying hens were acquired from a commercial flock (JSR Services Ltd.; Blairgowrie, UK) at 59 weeks of age. Hens consisted of experimental (*n* = 12) and companion (*n* = 7) birds. Three experimental birds could not be used post-surgery due to a failure in logging equipment and so are not referred to further. The remaining experimental birds made up experimental batches 1–3 (3 hens per batch). A further three hens were collected from the same commercial flock at 65 weeks of age to make up batch 4. Upon arrival, all birds were weighed, were wing-tagged with Ketchum wing tags (Putham, UK) and were placed in the holding room.

### 2.2. Housing

Birds were housed in two different rooms: one for general holding and one for experimental treatments. Both rooms were similar in size, had concrete floors, had LED lighting (one light above each pen) and were automatically heated and ventilated to maintain the room at 21 °C. Lights were on daily from 05:00 to 19:00 GMT (14L:10D) to reflect a standard commercial egg production environment. Pens in both rooms were made of wooden frames with an area of 2 m^2^ each and wire mesh siding (5 × 5 cm) that was 1 m high. A layer of fine wire mesh (1 × 1 cm) was added up to a height of 0.5 m with the pen walls above 1 m and made of plastic mesh (5 × 12 cm). Pen floors were filled with wood shavings as litter. Birds were provided with ad libitum access to layer pellets (16% crude protein and 11.5 MJ energy; ForFarmers UK Ltd., Rougham, UK) and water, approximately 1 kg of alfalfa hay used as enrichment (topped up as required), and a perch 60 cm long × 40 cm high was used.

#### 2.2.1. Holding Room

**Figure 1:**
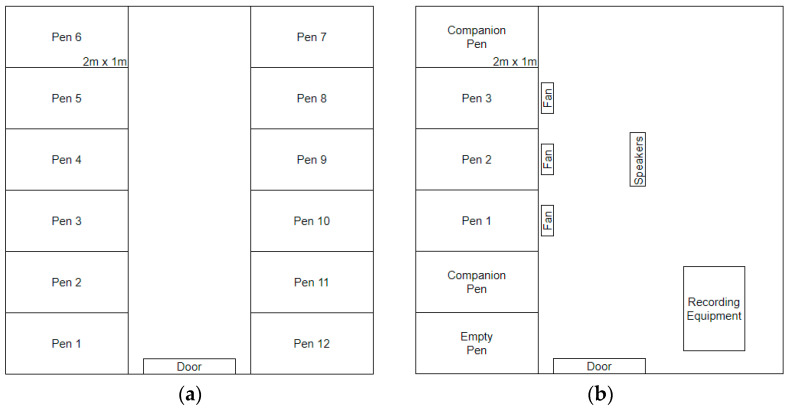
(**a**) Holding room layout and (**b**) experimental room layout. Experimental birds were in groups of 3 (e.g., birds 1–3, birds 4–6, etc.) at a time.

The holding room (Figure 1a) consisted of 12 pens (6 pens on either side of the room) that were 2 m deep × 1 m wide and made with wire mesh and wood frames. Here, floor pen litter was approximately 5 cm deep, and hens also had access to nest boxes (1 nest box per pen). The first 19 hens were housed in 7 pens of 2 birds each and 5 pens of a single bird each. Birds were housed individually or in pairs based on their behaviour and signs of feather pecking to maximize welfare and comfort, and they always had visual and auditory contact with neighbouring birds. Bird weight and condition was used to determine which birds were to be used as experimental animals, with the lightest birds and those with the worst feather condition being used as companion animals. As the total number of birds in the holding room decreased, paired birds identified as experimental hens were separated into individual pens. Birds in batch 4 were added after birds 1–3 had been moved to the experimental room.

#### 2.2.2. Experimental Room

Hens were housed in this room after EEG surgery to record their sleep behaviour. The experimental room (Figure 1b) consisted of five pens (all on one side of the room) of identical construction to those used in the holding room. The flooring of each pen was filled with approximately 2.5 cm of wood shavings to limit sitting/sleeping in the litter and to encourage a greater amount of perching. In these pens, hens had no nest boxes to allow cameras to record all of their behaviours, and perches were 30 cm long. One companion bird was always in each of the end pens, with the three focal experimental birds occupying the three central pens (one hen/pen). Companion birds were used to ensure that all experimental birds had a conspecific on either side with whom to remain in auditory and visual contact. Companion birds were replaced after the end of batches 1 and 3 to limit their exposure to experimental stressors (Table 1).

### 2.3. EEG Implants

**Figure 2:**
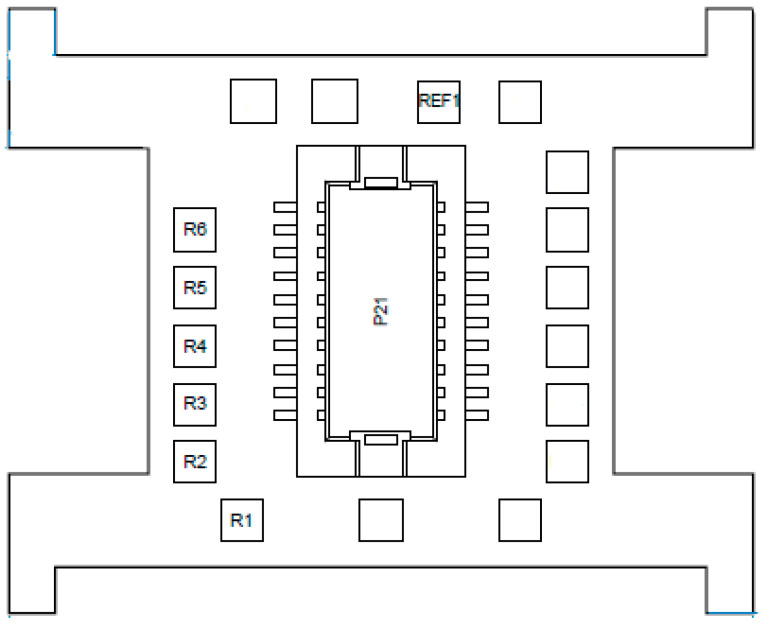
Electronic interface board (EIB) microchip schematic showing six referential channels for electroencephalograph (EEG) output (R1–R6) and one reference channel (REF1). P21 is the site in which the logger device was connected to the implant.

**Figure 3:**
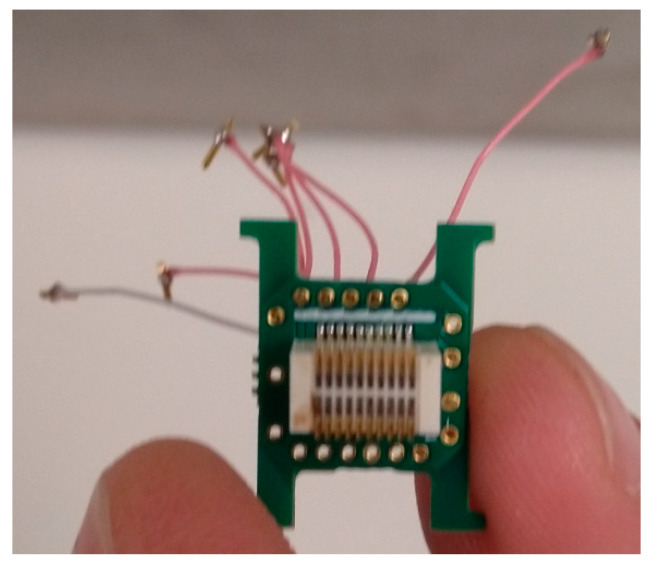
Implanted electronic interface board (EIB) microchip showing electroencephalograph (EEG) channel, reference channel wires and electrodes.

EEG implants were used to record brain activity during sleep. Implants consisted of an electronic interface board (EIB) microchip (ViewPoint Behavior Technology; Lyon, France). To transmit electrical brain activity to the chip, 0.25 mm diameter insulated wires were soldered to the EIB on one end and were soldered to a socket adaptor on the other. Six wires of 2.5 cm each were used for EEG probes, and one wire of 3.5 cm was used for the reference/baseline electrode. The number of wires used corresponded with the number of EEG channels to be used (Figure 2). Approximately 0.5 cm of each wire was exposed and wrapped around a 3 A, 100 V, IC socket adaptor (0.40 mm diameter and 2.5 mm length) (Bürklin Elektronik; Oberhaching, Germany). This adaptor was used as the electrode for measuring brain wave activity. The wire and IC socket adaptor were soldered together using soldering flux (Farnell; Paisley, UK); a 25 W, 230 V soldering iron (Farnell; Paisley, UK) and 1 mm solder (Maplin Professional Services; Edinburgh, UK). The opposite end of the wire was stripped by approximately 0.3 cm; the wire was then fed through the bottom of the correct hole (see Figure 3) and was unravelled. An EIB pin was positioned over the hole, and special pliers were used to press the pin into the hole to secure the wire (Figure 3). Frayed wire ends were removed with a scalpel blade to ensure accurate signal conductance and were covered in a thin layer of dental acrylic (Kemdent; Swindon, UK) to prevent detachment of wires during surgical implantation. Probe ends were cleaned with acetone to disinfect them prior to implantation.

### 2.4. Surgical Preparation

All operating surfaces were made aseptic using Milton sterilising fluid (2% sodium hypochlorite in water; Milton International; Nantes, France); all surgical tools were washed and sterilised in sterilising fluid (from batch 1) and then via autoclave (from batch 3 onwards) prior to surgical procedures. Three birds (constituting one batch) were pre-selected for surgery 2–3 days prior to the surgery date. One bird (randomly selected to be first) was fasted overnight (approximately 12 h) before surgery. The remaining two birds were fasted on the surgical day starting at approximately 07:30.

### 2.5. Surgical Procedure

Birds were brought into the operating room one at a time, were weighed, and were placed inside a cage measuring 49 cm × 55 cm × 78 cm. Ketamine (Ketamidor, Chanelle; UK) at 5 mg/kg and butorphanol (Torbugesic, Covetrus; Portland, ME, USA) at 0.4 mg/kg were given intra-muscularly to act as a sedative and analgesic, respectively, prior to provision of anaesthesia. Anaesthesia was induced using 4–5% sevoflurane (Zoetis; Louvain-la-Neuve, Belgium) per 1 L O_2_ and was maintained at between 2 and 2.5%. Vital signs (i.e., pulse, body temperature and oxygen saturation) were continuously monitored during surgery; additionally, fluid levels were maintained with regular injections of saline solution via a brachial vein indwelling catheter. After confirming complete anaesthesia using comb and toe pinches, birds were placed in a stereotaxic frame, with the head secured using ear bars. Birds were placed on top of a fluid-absorbing pad to collect excreta as well as a self-heating pad and covered in a fleece blanket to maintain body temperature during surgery.

Pet clippers (Langba; Ningbo, China) were used to remove feathers on the head within an approximately 4.5 cm × 4.5 cm area; any larger remaining feathers were removed with forceps. The area was cleaned with hydrogen peroxide prior to incision. A single incision was made using a Number 10 scalpel blade (Swann–Morton; Sheffield, UK), running from approximately 0.2 cm posterior to the comb to approximately 0.5 cm anterior to the neck musculature. From here, four haemostat clamps (Surtex Instruments Ltd.; London, UK) were attached to the four corners of the incision site to hold the skin open and to expose the cranium. Dry cotton swabs were used to remove the connective tissue on top of the cranium as well as to absorb any blood. Hydrogen peroxide was used to further clean the skull and to highlight any remaining material. The cranium was then dried.

The implant methodology used by [56] was employed. A point 1 cm anterior to the fusion lines of the cranial bones was demarcated as the point of origin using a skin marker (P^3^; Bristol, UK) and was used to delimit the length of the hyperpallial region. From here, dots were placed at 0.2 cm on either side of the origin to indicate the placement of the first two EEG probes. A second point was placed 0.7 cm anterior to the origin from where dots were placed at 0.3 cm on either side. A third point 0.3 cm anterior to the origin was demarcated, and a final two dots were placed at 0.4 cm on either side. This resulted in a total of 6 EEG probes with as wide a spread across the hyperpallium as possible. A final smaller ‘X’ was placed approximately 0.5 cm towards the bottom-right corner from the origin (in the porous bone) above the cerebellum to identify where the reference probe should be placed.

The demarcated dots were then drilled to expose the dura beneath. The tip of a scalpel blade was used to score all seven of the drill sites to prevent the drill bit from slipping during drilling. A 0.5 mm drill bit fitted to a manual pin vise drill (XLYYLWB; China) was used to drill the seven holes as well as to drill several anchor holes within the porous bone of the posterior part of the skull to act as additional surface area for the dental acrylic to adhere to. The drilling of the probe holes went only as far as the dura, which could be identified by a slight change in resistance and confirmed by a difference in hue from that of the cranial bones. From here, a low-viscosity drop of dental acrylic was placed in each probe hole immediately prior to probe insertion to ensure probes did not fall out of their designated holes. Upon securing all seven probes, a higher-viscosity dental acrylic was used to create a foundation across the probe ends protruding from the probe holes and across the anchor holes. The EIB microchip was then manipulated to be at a sufficient height (approximately 1 cm vertically from the skull) to allow for the probe wires to be tucked underneath, to prevent skin irritation, and to later allow for the EEG device to sit flush against the implant. From here, a thick layer of dental acrylic was steadily built up to encase the wiring underneath and to secure the EIB microchip to the skull.

Prior to completely encasing the bottom and corners of the microchip, two small omega-shaped metal hooks bent downwards at 90 degrees (handmade from large paperclips) were placed lateral to the implant and were secured with dental acrylic. Prior to suturing, 2% lidocaine (Hameln Pharmaceuticals; Gloucester, UK) was applied topically to the skin to minimize any immediate post-operative pain. 1 suture was placed anterior to the implant, and 1–2 sutures were placed posteriorly. After completion of surgery, birds were taken off anaesthesia and were monitored for signs of wakefulness (e.g., reflex test response, movement and independent breathing). Once muscle tone was present and once birds could stand, they were placed inside a second cage (identical to that used pre-surgery) with food, water, wood shavings as litter and a heat source if the bird was slow to wake. Once birds were alert and standing, they were brought to the experimental room to recover.

### 2.6. Post-Surgical Recovery and Monitoring

Birds were monitored during post-operative recovery for six days as well as being habituated to the experimental pens. General behavioural observations (e.g., body posture, activity levels and egg laying) and assessment of health were used as an overall guide. Birds were weighed daily to monitor their health and to determine the correct dosage for post-operative medications. The medications given were as follows: meloxicam (Meloxidyl, Covetrus; Portland, ME, USA) (1 mg/kg) was given subcutaneously twice per day as an analgesic for a total of 4 days; enrofloxacin (Baytril, Covetrus; Portland, ME, USA) (10 mg/kg) was given subcutaneously once per day as an antibiotic for a total of 6 days and dexamethasone (Dexafast, Covetrus; Portland, ME, USA) (0.5 mg/kg) was given intra-muscularly once per day to reduce the likelihood of neurological complications for a total of 2 days. Post-operative medications were provided in the morning at approximately 09:00 with second dosing (meloxicam only) provided at approximately 16:00.

### 2.7. EEG Devices

EEGs were monitored and recorded using the ONIEROS device (ViewPoint; Lyon, France). The device weighed 56 g (i.e., no more than 3.4% of body weight of the lightest bird used) and measured 3 cm × 2 cm × 1.5 cm. Each device was clicked into the implant and was further secured with a rubber band using the omega-shaped hooks. Devices were charged daily. Devices were removed at 08:30 every morning during routine husbandry and charged for 1 h (maximum charging time required) after which they were re-attached. This allowed for 23 h of continuous EEG recording for each bird.

### 2.8. Data Recording

EEG data were collected using EphyLab software (ViewPoint; Lyon, France). Each individual EEG channel, a reference channel, and three channels for accelerometery (*x*, *y* and *z* axes) were collectively recorded in real time and were automatically parcelled into four-hour-long data files.

**Figure 4:**
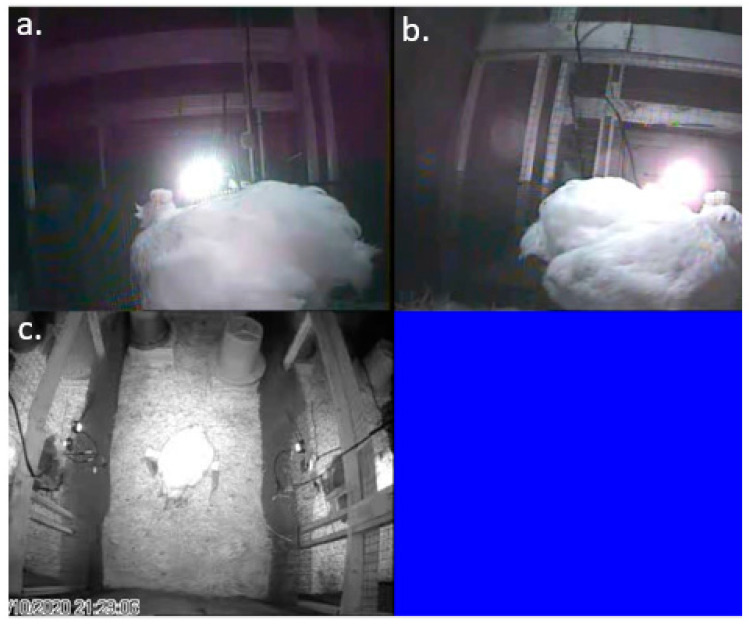
Camera framing for nighttime recording of (**a**) the left side of the bird, (**b**) the right side of the bird and (**c**) an overhead view of the pen with the bird perching.

Each experimental pen was outfitted with three infrared cameras: two H.264 CCTV IMX323 night vision IR mini cameras (Ailipu Technology Co., Ltd.; Shenzhen, China) on either side of the perch and a third high-resolution varifocal camera (Twilight CCTV; Merseyside, UK) pointing downwards from the top of the pen to capture the entire enclosure in frame (Figure 4). All nine cameras were connected to a computer with Geovision (Taipei, Taiwan) recording software installed which recorded and saved all video files in ‘.avi’ format. Nighttime recording of both EEGs and behaviour via video started at lights out (19:00) and ran until 08:30 the following day when devices were removed for charging. Daytime recording resumed at 09:30 and continued until 19:00. EEG and behavioural recordings were synchronized across devices.

Each experimental batch of hens was recorded for six 24 h periods. Recording periods consisted of undisturbed nights, disturbed nights and recovery nights (after a disturbed night) to observe both baseline and disruption effects on bird sleep behaviour. The allocation of disruptions per day per batch are given in Table 2. It was not possible to completely randomize the given nights, as a recovery night always had to follow a disturbed night. EEG devices would be attached to birds prior to lights out on the first night and then set to begin recording (in tandem with the three infrared cameras) at 19:00. Birds were manually placed on perches to ensure complete recording of sleep behaviours during the dark cycle. The total duration of the dark period was 10 h during which there was a 6-h span of sleep disruptors that were ordered to ensure balance (Table 3). Disruptors were set to turn on every half hour between 21:00 and 02:30, remain on for a duration of five minutes and then turn off again. The three disruptors used were wind from a small fan, noise generated by an mp3 player playing a traffic audio file on repeat and speakers set at a volume of 90 dB and light generated by the normal operating lights set at a maximum of 20 lux. Following a disturbed night, a recovery night would involve the use of the EEG devices and cameras without any of the disruptors. Undisturbed nights also used EEG devices and cameras without any of the disruptors and were used as a means of establishing a baseline for sleep behaviour. Devices were removed on the morning of the seventh day following recording.

### 2.9. Data Collection and Processing

EEG data were processed using Slip Analysis (ViewPoint; Lyon, France). Individual EEG files (noted by date and time) were uploaded to the software and were merged with the corresponding video files. Slow-wave sleep (SWS), rapid eye movement sleep (REM), wakefulness, and artefacts (electronic noise) were scored at the start of every minute using an epoch length of four seconds based on the amplitude of the EEG waves. SWS is identified by the presence of high-amplitude low-frequency waves, while both wakefulness and REM sleep are identified via low-amplitude high-frequency waves. Videos of hen behaviour were used as confirmation for intermediate (or uni-hemispheric) states or states that were more difficult to define based solely on EEG data such as between wakefulness and REM sleep (Table 4). The presence of one open eye was a clear indicator for instances of intermediate state, where wave amplitude alone was not sufficient. Additional behavioural indicators were used to discern REM sleep from wakefulness, closed eyes and drooping head being the primary indicators.

### 2.10. Statistical Analysis

Data were grouped into 2 h (lights off) or 3.5 h (lights on) time intervals in order to account for potential temporal effects. Intervals for birds in which the total proportion of missing data + artefact > 0.25 was omitted from further analysis after visual inspection of distributions. The counts for each sleep state were calculated for each bird in each interval from which proportions were calculated. Each 24 h period starting at lights off was classified by ‘type of night’, where experimental nights were categorized as either ‘disturbed’, ‘recovery’ or ‘undisturbed’ depending on whether disturbances were applied. In order to normalise residuals, proportions of sleep behaviours were angular transformed prior to fitting linear mixed models (LMMs). LMMs were applied separately for each sleep state and for lights off and lights on. Fixed effects were time interval, type of night and the interaction between them, and random effects were batch, bird, experimental day and time interval within experimental day. The reverse order of factors was also tested to ensure the results were similar. *p* values were based on approximate F tests using Satterthwaite and Kenward–Roger methods for denominator degrees of freedom. Estimates of means ± standard errors (SEs) obtained from the models were back transformed onto the proportion scale to aid interpretation. Mean values are presented with lower and upper bounds calculated through the subtraction of the SE from the mean (lower bound) or addition of the SE to the mean (upper bound), which was subsequently back transformed, and the least and greatest values identified. Post hoc tests between estimated means were carried out using Tukey’s HSD test. *p* values were compared against the standard of 0.05. Data processing and statistical analyses were carried out in the R system for statistical computing version 4.2.2 [71], which was accessed via RStudio 2022.10.0 Build 353 (RStudio Team, 2022).

## 3. Results

Of the 12 birds implanted with EEGs, 1 was culled due to meningeal and cerebral congestion, while a second lost the entire surgical implant 3 days after surgery and was culled as a result. The remaining 10 birds completed the trial with no adverse health effects and with the approximate maintenance of their body weight throughout the study (mean body weight change over 94 days of −52.5 ± 85.1 g). The scoring of the different sleep states resulted in a total of 65,468 observations across all the birds. After collating data at the level of sleep state and then subdividing the data into lights off (2 h intervals) and lights on (3.5 h intervals), there were 1530 observations across all the lights off periods and 720 observations across all the lights on periods. Where missing + artefact > 0.25 was removed, this resulted in the exclusion of 264 observations (as a proportion: 0.17) from the lights off data (*n* = 9) and 175 (0.24) observations from the lights on data (*n* = 10).

### 3.1. Lights Off

**Figure 5:**
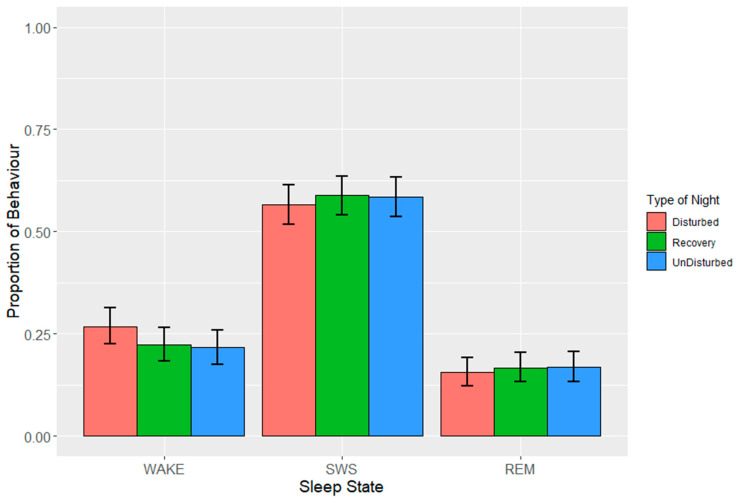
Effects during lights off of the three different types of night on proportions of states (wakefulness (WAKE), slow-wave sleep (SWS) and rapid eye movement (REM) sleep). Values are back-transformed means ± SEs estimated from LMMs fitted to angular transformed proportions.

During lights off, the observed mean proportions + the standard deviations (SDs) of the sleep behaviours over all the night types were 0.24 ± 0.08 (wakefulness), 0.58 ± 0.09 (SWS) and 0.18 ± 0.06 (REM sleep). Sleep behaviour on the undisturbed nights (i.e., baseline sleep behaviour) consisted of 0.25 ± 0.08 (wakefulness), 0.58 ± 0.08 (SWS) and 0.17 ± 0.03 (REM sleep). Resting behaviour was not observed during the lights off period. Averaged over the intervals within the night, the effect of the type of night was not statistically significant for any of the sleep states (Table 5). The estimates from the LMMs (Figure 5) suggested that the birds spent, on average, 0.22 to 0.27 of the night awake, 0.57 to 0.59 in SWS, and 0.16 to 0.17 in REM sleep over the different types of night.

There was a highly significant effect of the time interval on all the sleep states (Table 5). Averaging over the type-of-night effects, the post hoc tests showed that wakefulness was highest and that SWS was lowest between 03:00 and 05:00 (shortly before lights on at 05:00) compared to the other four intervals (*p* < 0.001) (Table 6), whereas REM sleep was lowest at 19:00–21:00 and then similarly high across the other four time intervals (*p* < 0.001). For REM sleep, only time interval was significant.

**Figure 6:**
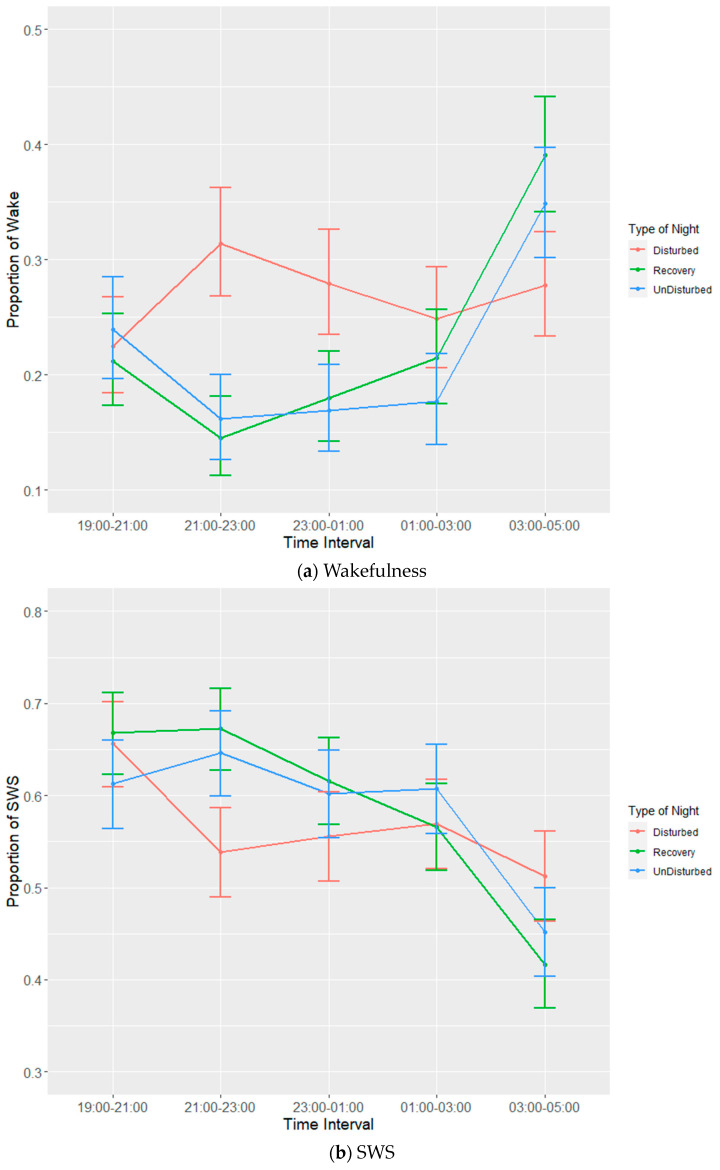

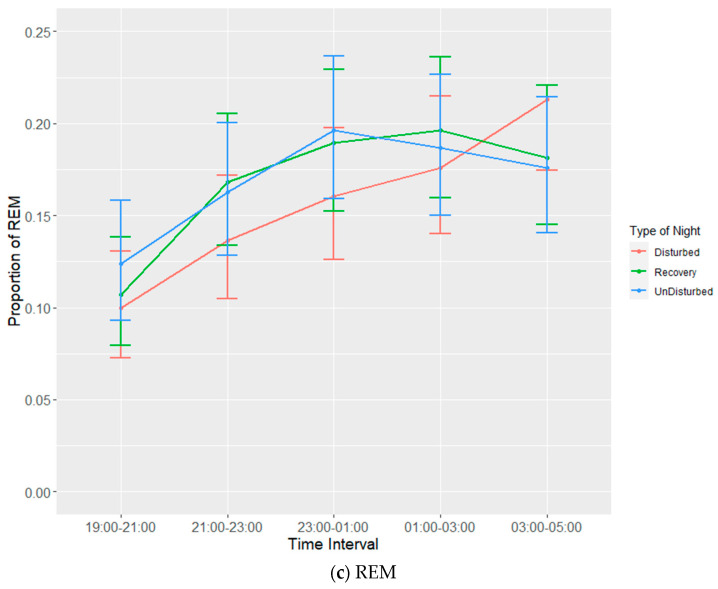
Effects during lights off of the interaction between time interval and three different types of night on proportions of states: (**a**) wakefulness (WAKE), (**b**) slow-wave sleep (SWS) and (**c**) rapid eye movement (REM) sleep. Values are back-transformed means ± SEs estimated from LMMs fitted to angular transformed proportions.

There was a significant interaction between the time interval and the type of night for wakefulness and SWS but not for REM sleep (Table 5, Figure 6a–c). The post hoc tests confirmed that the proportion of wakefulness was higher during 21:00–23:00 and 23:00–01:00 on disturbed nights compared to recovery and undisturbed nights during the same time intervals, whereas there was less wakefulness on disturbed nights at 03:00–05:00 (following the cessation of disturbances) compared to the recovery and undisturbed nights. The post hoc tests showed there was lower SWS during 21:00–23:00 during disturbed nights compared to recovery and undisturbed nights at the same time intervals, while SWS was higher during the final time period (03:00–05:00) during disturbed nights compared to recovery and undisturbed nights. SWS on disturbed nights was also marginally greater at 19:00–21:00 (when no disturbances had been applied yet) compared to at 03:00–05:00 (when disturbances had ceased) (*p* = 0.05); however, SWS did not differ between time periods on recovery and undisturbed nights.

### 3.2. Lights On

**Figure 7:**
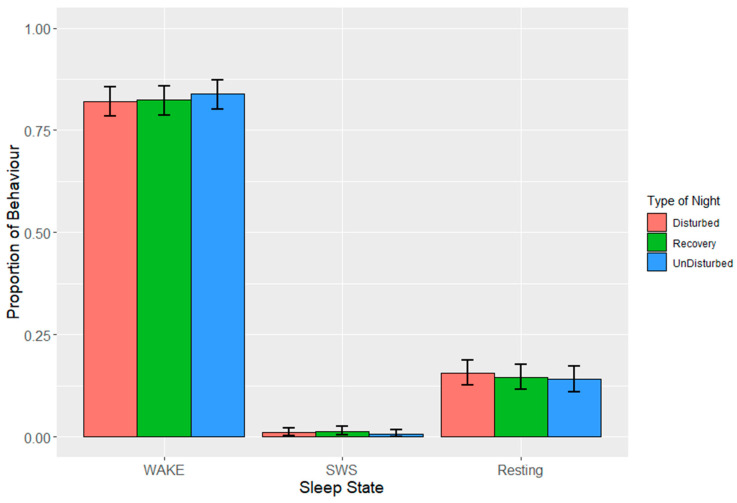
Effects during lights on following the three different types of night on proportions of states (wakefulness (WAKE), slow-wave sleep (SWS) and resting). Values are back-transformed means ± SEs estimated from LMMs fitted to angular transformed proportions.

**Figure 8:**
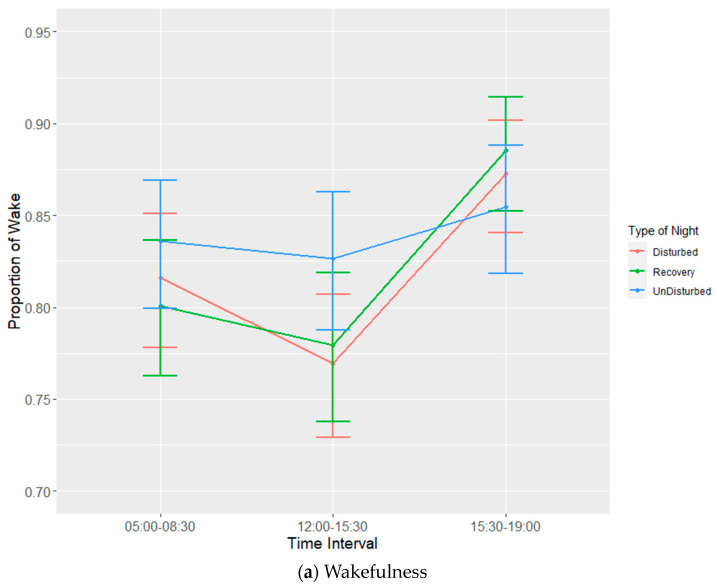

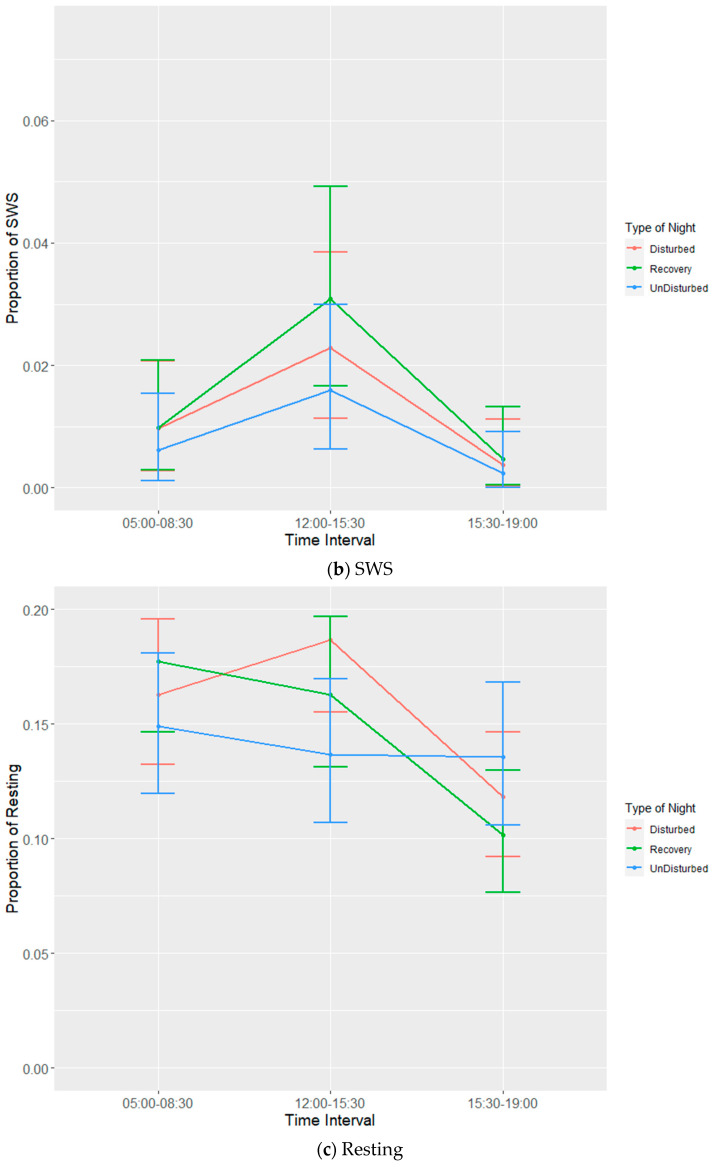
Effects during lights on of the interaction between time interval and three different types of night on proportions of states ((**a**) wakefulness (WAKE), (**b**) slow-wave sleep (SWS) and (**c**) resting). Values are back-transformed means ± SEs estimated from LMMs fitted to angular transformed proportions. There was no REM sleep behaviour observed during lights on.

During lights on, the observed mean proportions ± SDs of the sleep behaviours over all the night types were 0.81 ± 0.09 (wakefulness), 0.03 ± 0.04 (SWS) and 0.00 + 0.00 (REM sleep). The remaining 0.16 (± 0.06) was taken up by resting behaviour. The sleep behaviours following undisturbed nights (i.e., baseline sleep behaviour) were made up of 0.81 ± 0.09 (wakefulness), 0.02 ± 0.03 (SWS) and 0.17 ± 0.07 (resting). Estimates from the LMMs (Figure 7) suggested the birds spent, on average, 0.82 to 0.84 of the lights on period awake, 0.00 to 0.01 in SWS, and 0.14–0.16 of the lights on period resting during lights on following the various types of nights. The time interval significantly affected all three of these states (*p* ≤ 0.05), but there was neither a significant effect of the type of night on any sleep state during the subsequent lights on period nor an interaction effect (Table 7, Figure 8a–c).

During the lights on period, wakefulness was highest, and resting was lowest in the last period before lights off (15:30–19:00) (Table 8). There were only very small proportions of SWS observed, but SWS was highest in the middle of the lights on period.

## 4. Discussion

### 4.1. Lights Off

During periods of darkness, laying hens appeared to follow the general sleep trends for birds in that there was consistently more SWS than REM sleep and that, overall, SWS was greater at the beginning of the lights off period while REM sleep was greater towards the end of the lights off period [72,73]. Undisturbed nights were used to determine the baseline sleep characteristics of laying hens.

On average, laying hens spent approximately one quarter of the lights off period awake, over half of the night in SWS and the remaining time engaged in REM sleep. Pigeons have a somewhat similar sleep composition to that observed here [49,74], which is also evident in non-migratory sparrows [53]. However, with regards to sparrows kept on a light schedule similar to that of the hens used in this study, the time spent awake was only ~15%, SWS was ~57%, REM sleep was only 1% and the remaining 27% was attributed to drowsiness, which made up over 20% of total sleep time during the night [72]. Drowsiness is often considered an intermediate state between wakefulness and SWS, though, based on the behavioural and electrophysiological criteria used in this study, it would be classified as wakefulness, which would make the results found by Jones et al. [72] comparable to those found in laying hens [26].

For all the states observed, the type of night alone did not have any significant effects on sleep or wakefulness behaviours, but the interaction between the time interval and the type of night did affect wakefulness and SWS. This suggests that, while the disturbances themselves were effective at disturbing sleep within most of the time intervals in which they were applied, there was no effect of disturbances when the night was taken as a whole. This is apparent in the significantly higher amounts of wakefulness that were observed during the first 4 h of disturbance on disturbed nights compared to both recovery and undisturbed nights. Consequently, there was evidence of an attempt to make up for lost sleep on disturbance nights as seen in the significantly lower proportion of wakefulness and the higher proportion of SWS observed on those nights during the final 2 h before lights on. Notably, REM sleep was not significantly affected by these short-term disturbances.

In comparison, in subjecting sparrows to 6 h of continuous sleep deprivation followed immediately by 8 h of recovery sleep, their sleep behaviour was observed to change markedly: during recovery sleep, waking reduced from 100% (during deprivation) to ~20%, while SWS increased from 0% to a peak of almost 80% and while REM sleep moderately increased from 0% to a peak of around 4% [72]. Normally, non-migratory sparrows spend about 30% of the night awake, about 60% in SWS and the remaining 10% in REM sleep [53]. These findings are similar to what was observed in the present study. Zebra finches appear to be rather unlike pigeons, sparrows and laying hens as a study by Low et al. [75] observed a maximum of 50% of sleep in a night being made up of SWS, dropping to as low as 25% for the last several hours of the night. Consequently, there was an increase in REM sleep as the night progressed, going from as little as ~5% to as much as 35% [75]. Findings in zebra finches are not dissimilar from those seen in great frigatebirds (when sleeping on land), who spent ~45% of their total sleep time awake, 50% in SWS and about 5% in REM sleep [56]. Despite owls being a nocturnal species, they spend a similar amount of time, overall, in wakefulness and sleep behaviours as laying hens [76]. Ostriches have differing proportions of time spent asleep, with up to 20% of the night spent in REM sleep, though their REM sleep has been observed to overlap with SWS [40]. This is in contrast to pigeons and sparrows but is not much different than that observed in laying hens, suggesting an effect of a more terrestrial lifestyle on sleep requirements. There might also be an effect of body size, as both laying hens and ostriches are several times heavier than the small flying species outlined above. Overall, there appears to be a consistency in the sleep requirements of the avian species studied to date, though there are extreme situations, such as migration or sleeping on the wing, in which exceptions exist.

The lack of carry-over effects of sleep deprivation in this study in the days following disturbance or recovery nights could be attributed to the fact that the disturbances were applied for 5 min every 30 min (totalling only 1 h of disturbance in a single night) or to the fact that a 2 h undisturbed time interval immediately followed the final disturbances. This stands in contrast to the majority of studies dealing with sleep deprivation that tend towards depriving animals of sleep for several continuous hours [49,67,77,78]. In fact, Martinez-Gonzalez et al. [49] specifically designated an 8 h sleep deprivation period for pigeons as ‘short term’ sleep deprivation. In keeping with the behavioural definitions of sleep, the response to this is rebound sleep, resulting in an increase in either SWS or REM sleep intensity commensurate with the degree of deprivation [49,68,77,79,80,81,82,83,84,85,86]. While we did not record sleep intensity in this study, which may account for the lack of an effect seen on REM sleep, there appears to be an attempt between 03:00 and 05:00 by the hens to recover some lost sleep on disturbed nights given the significantly lower amount of wakefulness and the greater amount of SWS observed at that time. It is, therefore, possible that, while more significant bouts of sleep deprivation result in the typical increases in sleep intensity, smaller bouts are more manageable and can be dealt with via small increases in sleep duration.

### 4.2. Lights On

As with above, the baseline measures of sleep behaviour were determined solely using the days following undisturbed nights. On average, the laying hens spent 81% of the lights on period awake, approximately 3% in SWS, 0% in REM sleep and the remaining 16% of the lights on period engaged in resting behaviour. Only the time interval had a significant effect on the behavioural states during lights on; thus, contrary to what was seen during the lights off period, the presentation of sleep disturbances the previous night had no effect on sleep behaviour the following day. Generally, less time spent in wakefulness and an increase in SWS or resting behaviour would be expected in the face of carry-over effects from the previous night. Regardless of the type of night, there was an increase in wakefulness and a decrease in resting in the last lights on period observed. This may reflect a period of activity before lights off, possibly as hens engage in preparatory behaviours before sleep. Preparatory pre-sleep behaviours are often seen in birds prior to sleep/lights off, such as moving to roosting sites or feeding [64,86,87,88,89].

As expected from a diurnal species, laying hens spent the vast majority of the lights on period awake and active and only about 15% of the lights on period resting or sleeping. By comparison, a study by Alvino et al. [57] found that broilers spent about 20% of the day sleeping; however, these are juvenile animals and not adults, unlike the hens used in this study, which may account for greater daytime sleeping in broilers. A study in turkeys noted that sleeping occupied 15% of the daytime activity budget, but this steadily decreased to 0% within a 12-week period [20], perhaps as animals became mature. Similarly, Japanese quails were observed to spend 11% of the day resting [90]. More recent work in two different genotypes of Turkish laying hen found that hens spend about 40% of the day resting/inactive (here defined as a hen lying on its abdomen or sitting with its legs under its body), which is more than double that reported here for birds of the same age [91]. It is possible this discrepancy is due to Sözcü et al. [91] only recording the behaviour for two non-consecutive hours per day (9:00–10:00 and 15:00–16:00), the latter time interval partly intersecting with a time of day outlined above as consisting of a greater amount of rest. In contrast to poultry, Bäckman et al. [50] found that migratory shrikes spent an average of approximately 30% of the day resting, which increased in accordance with the time spent flying the previous night. It is apparent that the time that diurnal avian species allocate to resting is conditional on the satisfaction of their other needs (e.g., feeding and drinking), but this does not generally extend past 20% of all their daytime activities, which is in keeping with the findings of the present study.

The lack of significant effects of disturbance on behaviour during the lights on period is likely attributable to the findings noted above, namely, that, while the effects of the disturbances were effective at the time of application, the total degree of disturbance was likely too low to elicit any type of compensatory increase in sleep behaviour during the following day. This may also result from an increase in sleep intensity following the cessation of the disturbances, though this was beyond the scope of this study. Furthermore, the disturbances themselves were not applied during the lights on period. There was a noticeable increase in the time spent awake following both disturbed and recovery nights between 15:30 and 19:00. This increase was met with a complementary decrease in resting. Comparatively, SWS saw a small increase between 12:00 and 15:30 following all three kinds of night, most notably after recovery nights. Overall, this complementary behaviour in conjunction with a lack of treatment effects on behaviour is indicative of a natural preparatory period prior to sleeping.

### 4.3. Behavioural Indicators of Sleep

Studies have attempted to investigate sleep in a variety of species, often using purely behavioural measures, such as a lying posture or the time spent lying, as proxies. To date, this is the first study to observe the detailed sleep behaviours in laying hens using EEGs; however, it is acknowledged that this is not a practical solution to studying the sleep of laying hens in a commercial environment. Therefore, correlating behaviour with EEGs may be a better long term solution to studying sleep in commercial poultry.

It is possible that the use of behavioural correlates to identify sleep states may not be as easy to elucidate in all species. For example, studies in dairy cows have resulted in disagreements as to whether posture is a reliable indicator of sleep state [92,93,94]. Furthermore, these studies do not validate behavioural observations such as posture with electrophysiological data, leading to these measures being more akin to time spent lying down rather than accurate assessments of sleep state. However, having now used EEG data to corroborate measures of behaviour in this study, it may now be possible to move to fully behavioural assessments of sleep in laying hens without the need for invasive surgical procedures, using these characteristics as behavioural correlates of sleep state.

### 4.4. Sleep Behaviour as a Welfare Indicator

The commercial farm environment can be a challenging one; therefore, assessing and ensuring positive welfare should be a priority. Welfare state is more readily identifiable during regular daytime operating hours, and, indeed, it is this period of time that is often studied when assessing behaviour. However, it cannot be assumed that conditions that are satisfactory during the day are equally so at night, especially considering that all major farm animal species are diurnal. The requirements for sleep in vertebrate species are clear, yet sleep is frequently overlooked when assessing welfare. An examples of this includes the lighting programs used in broiler houses which, outside of Europe, are often on continuously or nearly continuously in order to encourage feeding behaviour and growth (e.g., [18]). Furthermore, studies looking at the effects of temperature have noted that variations in broiler health and welfare are closely linked to the temperature and humidity in their housing environment [95]. Body temperature increases in accordance with sleep (e.g., [96]), thus making sleep very likely to be affected by poor ventilation and temperature regulation conditions. Ultimately, sleep forms a critical component of the biological needs and behavioural repertoire of all animals studied to date and, therefore, offers a deeper insight into the overall welfare of the animal, especially when assessing the welfare of commercial animals via behaviour.

## 5. Conclusions

This was a first step in understanding the effects of various types of sleep disruptors on laying hens, and these findings provide further argument for the assessment of the complete behavioural repertoire, especially when considering the ability of an animal to be able to perform requisite behaviours as being a critical tenet of good welfare. The use of physiological measurements in the initial quantification of sleep behaviour, while invasive, allowed for the definitive identification of sleep while also opening the door for future work to use a strictly behavioural framework to study the effects of disturbances and sleep deprivation, albeit with a reduced degree of certainty. Further work should endeavour to observe the effects of more intensive commercially relevant disturbances, such as feed deprivation, thermal stress or pain. Additionally, extending the duration and timing of the sleep disruptions at night to determine if there are any carry-over effects of sleep deprivation as well as including measures of sleep intensity would be of interest. The findings of this study have improved our understanding of baseline sleep behaviour in poultry. These findings also provide a potential argument for the inclusion and use of sleep behaviour (given the sensitivity of sleep to disturbance) as an indicator of welfare.

## Figures and Tables

**Table 1:** Experimental and companion bird allocation according to their ID numbers and batch.

Batch	Companion Bird ID Numbers	Experimental Bird ID Numbers
1	1, 2	1, 2, 3
2	3, 4	4, 5, 6
3	3, 4	7, 8, 9
4	5, 6	10, 11, 12

**Table 2:** Experiment diary.

Day	Order of Actions 2 (Batch 1 and 3)	Order of Actions 1 (Batch 2 and 4)
Mon (Day −6)	Surgery	Surgery
Tues-Sun (Day −5–0)	Recovery, monitoring and habituation	Recovery, monitoring and habituation
Mon (Day 1)	Undisturbed sleep recording	Disturbed sleep recording
Tues (Day 2)	Disturbed sleep recording	Recovery sleep recording
Wed (Day 3)	Recovery sleep recording	Undisturbed sleep recording
Thurs (Day 4)	Undisturbed sleep recording	Disturbed sleep recording
Fri (Day 5)	Disturbed sleep recording	Recovery sleep recording
Sat (Day 6)	Recovery sleep recording	Undisturbed sleep recording
Sun (Day 7)	Equipment switched off	Equipment switched off

**Table 3:** Schedule for sleep disruptor application (incomplete Latin square) during lights off. Each disruptor was applied for 5 min each at the time shown (W = wind, L = light and N = noise).

Time	Batch 1	Batch 2	Batch 3	Batch 4
19:00–21:00	Undisturbed sleep			
21:00	L	N	L	W
21:30	W	L	W	N
22:00	N	W	N	L
22:30	W	L	W	N
23:00	L	N	N	W
23:30	N	W	L	L
00:00	W	N	N	L
00:30	L	W	L	N
01:00	N	L	W	W
01:30	L	N	L	W
02:00	N	W	W	L
02:30	W	L	N	N
03:00–05:00	Undisturbed sleep			

**Table 4:** Ethogram of sleep behaviour assessment criteria and corresponding electroencephalograph (EEG) patterns.

Video	EEG	Definition
Awake	Waking EEG (low-amplitude high-frequency waves)	Bird is clearly awake and engaged in activity, including walking, preening, nest building, laying, feeding, drinking or foraging.
Resting	Waking EEG (low-amplitude high-frequency waves)	Bird appears to be awake as evidenced by minor and infrequent head movements and may be in a stereotypic sleep posture (sitting with wings folded or head retracted into the breast) and not engaged in any active activity. One or both eyes may be closed with occasional opening. No corresponding EEG activity to suggest it is asleep.
Sleep	Slow-wave sleep (SWS) EEG (high-amplitude low-frequency waves)	Bird is in a stereotypic sleep posture (sitting or perching with wings folded and head retracted) with one or both eyes closed. EEG has transitioned from waking to SWS.
Sleep	Rapid eye movement (REM) sleep EEG (low-amplitude high-frequency waves—must be preceded by SWS)	Bird is in a stereotypic REM sleep posture (sitting or perching with wings relaxed and head hanging downwards) with both eyes closed. EEG has transitioned from SWS to REM sleep.

**Table 5:** *F* tests for effects of time interval (‘Time’, 2 h blocks), type of night (‘ToN’; disturbed, recovery and undisturbed) and their interaction (Time × ToN) on wakefulness, slow-wave sleep (SWS) and rapid eye movement (REM) sleep during lights off from LMMs. *F* values, with numerator and denominator degrees of freedom (ndf,ddf, respectively), are shown.

	Wakefulness			SWS			REM Sleep		
	Time	ToN	Time × ToN	Time	ToN	Time × ToN	Time	ToN	Time × ToN
ndf,ddf	4,49	2,10	8,50	4,47	2,10	8,47	4,177	2,12	8,177
F value	12.2	2.15	4.55	18.15	0.42	2.66	13.06	0.53	0.87
*p* value	< 0.001	0.167	< 0.001	< 0.001	0.669	0.017	< 0.001	0.600	0.540

**Table 6:** Effects during lights off of time interval on proportions of states (wakefulness, slow-wave sleep (SWS) and rapid eye movement (REM) sleep). Values are back-transformed means, with back-transformed mean − SE and back-transformed mean + SE forming the lower and upper bounds, respectively, estimated from LMMs fitted to angular transformed proportions. Where superscripts are different within a column, means are significantly different (*p* < 0.05) (obtained through post hoc Tukey’s HSD tests).

	Wakefulness	Lower Bound	Upper Bound	SWS	Lower Bound	Upper Bound	REM Sleep	Lower Bound	Upper Bound
19:00–21:00	0.22 ^b^	0.19	0.26	0.65 ^a^	0.61	0.68	0.11 ^b^	0.08	0.14
21:00–23:00	0.20 ^b^	0.17	0.24	0.62 ^a^	0.58	0.66	0.16 ^a^	0.13	0.19
23:00–01:00	0.21 ^b^	0.18	0.24	0.59 ^a^	0.55	0.63	0.18 ^a^	0.15	0.22
01:00–03:00	0.21 ^b^	0.18	0.25	0.58 ^a^	0.54	0.62	0.19 ^a^	0.15	0.22
03:00–05:00	0.34 ^a^	0.30	0.38	0.46 ^b^	0.42	0.50	0.19 ^a^	0.16	0.23

**Table 7:** *F* tests for effects of time interval (‘Time’, 3.5 h blocks), type of night (‘ToN’; disturbed, recovery and undisturbed) and their interaction (Time × ToN) on wakefulness, slow-wave sleep (SWS) and resting during lights on from LMMs. *F* values and numerator and denominator degrees of freedom (ndf,ddf, respectively) are shown.

	Wakefulness			SWS			Resting		
	Time	ToN	Time × ToN	Time	ToN	Time × ToN	Time	ToN	Time × ToN
ndf,ddf	2,28	2,13	4,28	2,118	2,118	4,118	2,28	2,15	4,28
*F* value	8.80	0.45	0.89	11.39	1.11	0.14	4.96	0.26	1.02
*p* value	0.001	0.647	0.484	< 0.001	0.334	0.968	0.014	0.771	0.412

**Table 8:** Effects during lights on of time interval on proportions of states (wakefulness, slow-wave sleep (SWS) and resting behaviour). Values are back-transformed means, with back-transformed mean − SE and back-transformed mean + SE forming the lower and upper bounds, estimated from LMMs fitted to angular transformed proportions. Where superscripts are different within column, means are significantly different (*p* < 0.05) (obtained through post hoc Tukey’s HSD tests).

	Wakefulness	Lower Bound	Upper Bound	SWS	Lower Bound	Upper Bound	Resting	Lower Bound	Upper Bound
05:00–08:30	0.82 ^b^	0.79	0.85	0.01 ^b^	0.00	0.02	0.16 ^a^	0.14	0.19
12:00–15:30	0.79 ^b^	0.76	0.82	0.02 ^a^	0.01	0.04	0.16 ^a^	0.14	0.19
15:30–19:00	0.87 ^a^	0.84	0.90	0.00 ^b^	0.00	0.01	0.12 ^b^	0.10	0.14

## Data Availability

The data sets collected, generated and analysed for this study are available from the corresponding author via Zenodo repository (6557169).
